# Plasma Neurofilament Light (NfL) in Patients Affected by Niemann–Pick Type C Disease (NPCD)

**DOI:** 10.3390/jcm10204796

**Published:** 2021-10-19

**Authors:** Andrea Dardis, Eleonora Pavan, Martina Fabris, Rosalia Maria Da Riol, Annalisa Sechi, Agata Fiumara, Lucia Santoro, Maximiliano Ormazabal, Romina Milanic, Stefania Zampieri, Jessica Biasizzo, Maurizio Scarpa

**Affiliations:** 1 Regional Coordinator Centre for Rare Diseases, University Hospital of Udine, 33100 Udine, Italy; pavan.eleonora@gmail.com (E.P.); rosalia.dariol@asufc.sanita.fvg.itr (R.M.D.R.); annalisa.sechi@asufc.sanita.fvg.it (A.S.); maxi.ormazabal@gmail.com (M.O.); stefania.zampieri@asufc.sanita.fvg.it (S.Z.); maurizio.scarpa@asufc.sanita.fvg.it (M.S.); 2 Institute of Clinical Pathology, Department of Laboratory Medicine, University Hospital of Udine, 33100 Udine, Italy; martina.fabris@asufc.sanita.fvg.it (M.F.); romina.milanic@asufc.sanita.fvg.it (R.M.); jessica.biasizzo@asufc.sanita.fvg.it (J.B.); 3 Regional Referral Center for Inherited Metabolic Disease, Department of Pediatrics, University of Catania, 95131 Catania, Italy; agatafiumara@yahoo.it; 4 Division of Pediatrics, Department of Clinical Sciences, Polytechnic University of Marche, Ospedali Riuniti, 60123 Ancona, Italy; dott.luciasantoro@gmail.com

**Keywords:** Niemann–Pick C, neurofilament light, biomarkers, neurological disease

## Abstract

(1) Background: Niemann–Pick type C disease (NPCD) is an autosomal recessive lysosomal storage disorder caused by mutations in the NPC1 or NPC2 genes. The clinical presentation is characterized by visceral and neurological involvement. Apart from a small group of patients presenting a severe perinatal form, all patients develop progressive and fatal neurological disease with an extremely variable age of onset. Different biomarkers have been identified; however, they poorly correlate with neurological disease. In this study we assessed the possible role of plasma NfL as a neurological disease-associated biomarker in NPCD. (2) Methods: Plasma NfL levels were measured in 75 healthy controls and 26 patients affected by NPCD (24 NPC1 and 2 NPC2; 39 samples). (3) Results: Plasma NfL levels in healthy controls correlated with age and were significantly lower in pediatric patients as compared to adult subjects (*p* = 0.0017). In both pediatric and adult NPCD patients, the plasma levels of NfL were significantly higher than in age-matched controls (*p* < 0.0001). Most importantly, plasma NfL levels in NPCD patients with neurological involvement were significantly higher than the levels found in patients free of neurological signs at the time of sampling, both in the pediatric and the adult group (*p* = 0.0076; *p* = 0.0032, respectively). Furthermore, in adults the NfL levels in non-neurological patients were comparable with those found in age-matched controls. No correlations between plasma NfL levels and NPCD patient age at sampling or plasma levels of cholestan 3β-5α-6β-triol were found. (4) Conclusions: These data suggest a promising role of plasma NfL as a possible neurological disease-associated biomarker in NPCD.

## 1. Introduction

Niemann–Pick type C disease (NPCD-MIM 257220; MIM607625) is an autosomal recessive neurovisceral lysosomal storage disorder due to mutations in the NPC1 (95% of patients) or NPC2 genes, encoding two proteins involved in the intracellular trafficking of cholesterol and other lipids. The deficiency of either of them leads to the accumulation of endocytosed unesterified cholesterol and other lipids within the lysosome/late endosome compartment [1].

The clinical presentation of the disease is extremely variable and the age at onset ranges from the perinatal period to adulthood. The disease is typically characterized by visceral and neurological signs and symptoms that follow an independent clinical course. When visceral involvement is present, it is characterized by hepatosplenomegaly that precedes the onset of neurological signs. Apart from a small group of patients presenting a severe perinatal form leading to death due to liver or respiratory failure within the first months of age, and a few mildly affected adult cases, all patients develop progressive and fatal neurological disease [2,3,4]. Indeed, NPCD has been classically classified on the basis of age at onset of neurological symptoms, irrespective of the age of first visceral symptoms [1].

Miglustat, a glucosylceramide synthase inhibitor, is the only therapeutic option approved for the treatment of neurological symptoms of NPCD. However, other approaches such us the use of arimoclomol (a co-inducer of the heat shock response) and 2-hydroxypropyl-β-cyclodextrin (HP-β-CD) are currently under clinical investigation [5]. In addition, a recent study supports the efficacy and safety of Tanganil (N-acetyl-L-Leucine) for the symptomatic treatment of NPCD [6].

To date, several molecules have been proposed and validated as biomarkers of NPCD to support the diagnosis and follow up of affected patients [7,8,9,10,11,12,13,14,15,16,17,18,19]. Among them, the plasma levels of cholestan-3β,5α,6β-triol (3β,5α,6β-triol) and 7-ketocholesterol (7-KC) are the most widely used in clinical settings [13,14,15,16,17,18,19]. However, a relatively weak correlation with severity or age at onset of neurological involvement has been found [13,20]. Therefore, new biomarkers that better correlate with neurological involvement, which represents the most devastating aspect of the disease, are needed.

Neurofilaments are major structural elements of neurons. They are composed of four subunits: a triplet of NF light (NfL), NF medium (NfM) and NF heavy (NfH) chains, and either α-internexin or peripherin in the central and peripheral nervous systems, respectively [21]. Nfs can be detected in the cerebrospinal fluid (CSL) as surrogate markers of axonal injury, degeneration, and loss [22]. As such, increased levels of NfL, the most abundant subunit, have been reported in the CSF of patients affected by several neurological diseases [23,24,25,26,27,28].

Although NfL has not been systematically studied in NPCD patients, elevated levels in CSF have been described in two case report studies [29,30].

Recently, highly sensitive assays have been developed for the detection of NfL in blood. Most importantly, a strong correlation between serum and CSF NfL levels has been demonstrated in several disorders [24,31,32,33,34,35,36,37,38].

Based on this evidence, plasma levels of NfL have already been investigated and found to be elevated in patients affected by two neurodegenerative lysosomal disorders: mucopolysaccharidosis II and neuronal ceroid lipofuscinosis type 2 [39,40].

Given the reports of elevated NfL levels in CSF or plasma in genetic, inflammatory, and other neurodegenerative diseases [23,24,25,26,27,28,29,30,31,32,33,34,35,36,37,38,39,40], NfL in body fluids appears to be a general, not disease-specific, but sensitive marker of neurodegeneration due to the loss of neurons and axons. This has led to the present study on the possible role of NfL in plasma as a biomarker for the neurological disease associated with NPCD.

## 2. Materials and Methods

### 2.1. Patients

Plasma samples from patients affected by NPCD were obtained during the diagnostic work-up at the Regional Coordinator Centre for Rare Diseases. Patient phenotype classification was based on the age at onset of neurological symptoms as follows: severe infantile (SI, age at onset <2 years), late infantile (LI, age at onset 2–6 years), juvenile (J, age at onset 6–15 years), and adult (A, age at onset >15 years) [41]. Patients who died during the first 6 month of life due to liver or respiratory insufficiency without signs of neurological involvement were classified as having the early infantile systemic lethal form (EISL). Asymptomatic patients and patients who did not present clinical or imaging signs of neurological involvement at last follow-up were considered non-classifiable (NC).

Control samples were obtained from 75 healthy age-matched subjects. Adult samples were obtained from healthy blood donors, while pediatric samples were recovered from material used for routine investigations, without further sampling, of children with normal neurological and liver function. The study was conducted in accordance with the ethical standards of 1964 Helsinki Declaration and its later amendments and written informed consent was obtained from all subjects or care/guardians on behalf of the minors.

NPCD diagnosis was established by NPC1 and NPC2 genotyping. Biochemical confirmation by the analysis of intracellular cholesterol accumulation by filipin staining was done in all patients presenting at least 1 allele carrying a variant of unknown significance (VUS) and whenever available in cases with pathogenetic variants of both alleles [42] (Table S1). Patients presenting at least 1 VUS and a variant biochemical phenotype presented high levels of NPCD biomarkers: oxysterols and/or N-palmitoyl-O-phosphocholineserine (PPCS). Genotype was confirmed in relatives whenever possible.

### 2.2. Neurofilament Light (NfL)

Plasma NfL was assessed using a microfluidic Simple PlexTM NfL Assay (ProteinSimple, San José, CA, USA) on an EllaTM instrument according to the manufacturers’ instructions. The instrument was calibrated using the in-cartridge factory standard curve. All samples were measured after a single thaw, with a 1:2 dilution.

### 2.3. Plasma Cholestane-3β,5α,6β-triol Concentration

The concentration of cholestane-3β,5α,6β-triol was determined as previously described [16]. Briefly, internal standard/protein precipitation solution (250 μL) was added to 50 μL of samples. After centrifugation, the supernatant was dried under a stream of nitrogen and then derivatized with N,N-dimethyilglycine (DMG) in the presence of both 1-ethyl-3-(3-dimethylaminopropyl)cabodiimide (ECD) and 4-(N,N-dimethylaminopyrididine) (DMAP). After quenching the reaction with 20 μL of methanol, tubes were dried with nitrogen stream and reconstituted with 200 μL of methanol–water (4:1). Sample analysis was performed by HPLC-MS/MS using a Prominence UFLCXR system (Shimadzu Scientific Instruments, Columbia, MD, USA) and a 4000 Qtrap MASS SPECTROMETER (ABSciex, Framingham, MA, USA).

### 2.4. Statistical Analysis

Descriptive statistics were used to describe the patients’ demographic and clinical characteristics at the time of sampling.

The nonparametric Mann–Whitney test was used to assess the significance of plasma NfL differences between groups. Relationships between plasma NfL and oxysterols or age were evaluated by Pearson’s correlation test. Analysis was performed using the GraphPad Prism v8 (GraphPad Soft-ware, San Diego, CA, USA).

## 3. Results

Plasma NfL levels were measured in 75 healthy controls and 26 patients affected by NPCD (24 NPC1 and 2 NPC2). Patients were classified according to the age at onset of neurological symptoms as previously described [1]. Serial samples obtained during regular follow-up visits were available for 8/26 NPCD patients. In total, 39 NPCD samples were analyzed. Details on the clinical phenotype, age at NfL assessment, and neurological involvement at the time of NfL assessment are summarized in Table 1 and Table S1. In total, 3 patients died during follow up: 2 of them, affected by the EISL phenotype (NP1 and NP2; Table S1), died from respiratory insufficiency and liver failure, respectively, before 6 months of age; and 1, affected by the EI clinical form, died from neurological disease at the age of 3 years (NP4; Table S1).

**Figure 1:**
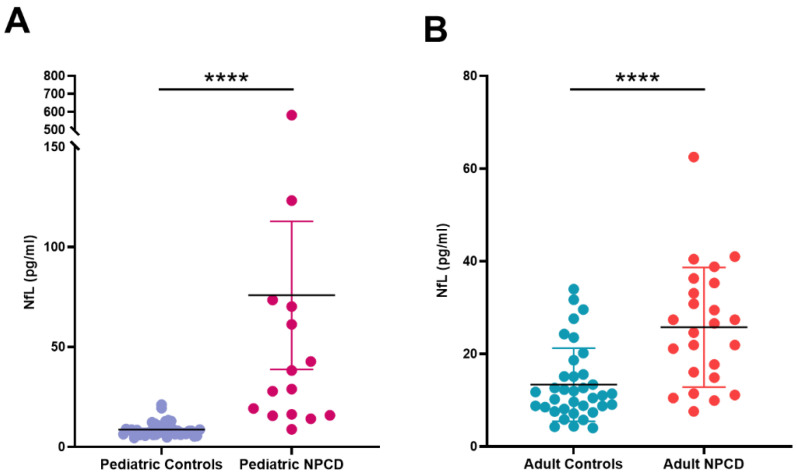
NfL concentrations in plasma of NPCD patients. (**A**) Pediatric NPCD patients vs. healthy controls. (**B**) Adult NPCD patients vs. healthy controls. **** *p* < 0.0001.

As already reported [43], plasma NfL levels in healthy controls correlated with age (Pearson’s correlation coefficient = 0.4313; *p* = 0.0097) and were significantly lower in the pediatric population (<18 years of age; *n* = 39; median: 7.94 pg/mL; IQR: 6.4–9.8 pg/mL) than in adults (≥18 years; *n* = 36; median: 11.2 pg/mL; IQR: 8.3–15.3 pg/mL) *p* = 0.0017. Therefore, to compare the NfL levels found in NPCD patients vs. healthy controls, samples were divided in 2 groups according to the age at the time of NfL assessment: <18 years of age and ≥18 years of age. As shown in Table 2 and Figure 1, in both pediatric and adult NPCD patients the plasma levels of NfL were significantly higher when compared with age-matched controls (*p* < 0.0001).

**Figure 2:**
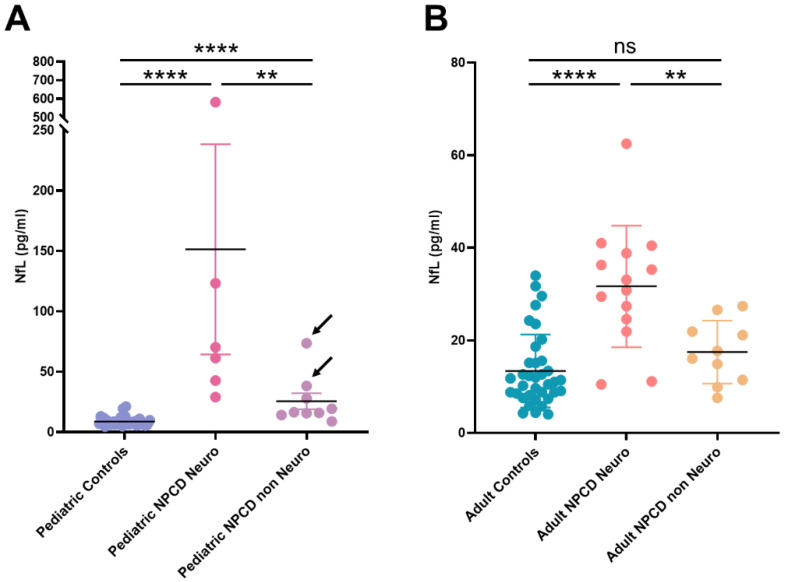
NfL concentration in plasma from neurological and non-neurological NPCD patients. (**A**) Pediatric patients. Arrows indicate NPCD patients affected by the EISL phenotype. (**B**) Adult patients. **** *p* < 0.0001; ** *p* < 0.01; NS: non-significant.

However, since 10/24 adult and 9/15 pediatric samples were obtained from NPCD patients free of neurological signs and symptoms at the time of sampling (Table S1), we divided both groups according to the presence or absence of neurological involvement and compared their NfL levels. As shown in Figure 2, NfL levels in NPCD patients with neurological involvement were significantly higher than the levels found in non-neurological patients, both in the pediatric and the adult group (*p* = 0.0076; *p* = 0.0032, respectively). Furthermore, in adults the NfL levels found in non-neurological patients were comparable with those found in age matched healthy subjects. Among pediatric NPCD patients, only two non-neurological patients displayed NfL values that overlap with those found in neurological patients (Figure 2). It is worth noting that these two patients were affected by the EISL clinical form, an extremely severe phenotype rapidly leading to death due to liver failure and respiratory insufficiency, before the development of neurological disease.

Since a possible bias in the presented results might have been introduced due to the inclusion of NfL values obtained in serial samples of the same patient in the NPCD group, we performed the analysis considering only one sample per patient (obtained during the first visit). As shown in Table 3, also excluding serial samples from the analysis, both pediatric and adult NPCD patients displayed significant increased levels of NfL when compared with age-matched controls, and patients with neurological involvement presented significantly higher levels of plasma NfL than non-neurological patients.

In two neurological patients (NP8 and NP21; Table S1), plasma NfL was assessed just after starting treatment with miglustat, while in other two neurological adult patients, plasma NfL were measured before and after starting miglustat therapy (NP18; NP18sib). In these last patients, plasma NfL levels remained stable or slightly decreased (Table S1).

**Figure 3:**
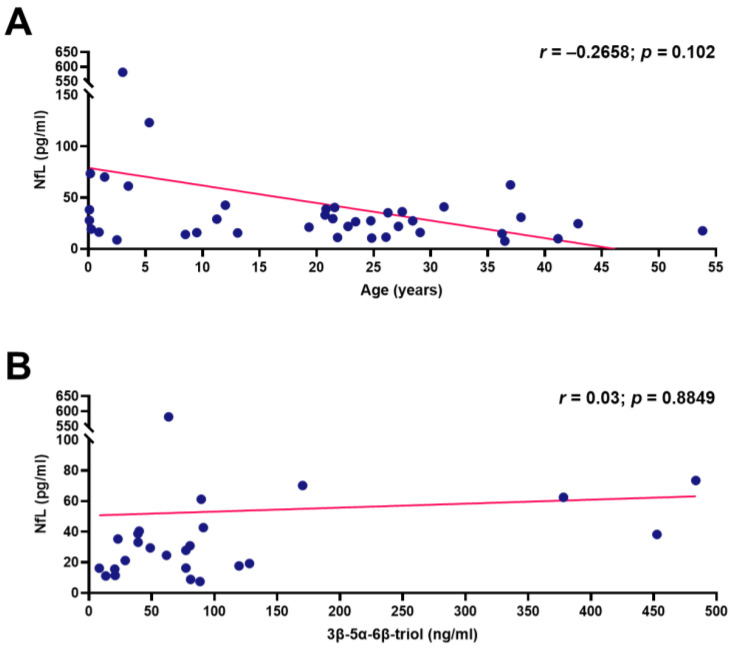
Association of NfL concentrations in the plasma of NPCD patients with age (**A**) and 3β,5α,6β-triol (**B**). Correlations were assessed by Pearson’s linear regression correlation test.

In contrast to healthy controls, no correlation between plasma NfL levels and age at sampling was found in NPCD patients (Pearson’s correlation coefficient = −0.2658; *p* = 0.102) (Figure 3A).

Plasma levels of cholestan 3β-5α-6β-triol were measured in samples taken during the first visit in 25 out of 26 patients, and no correlation between plasma levels of this oxysterol and those of NfL was found (Pearson’s correlation coefficient = 0.03; *p* = 0.8849) (Figure 3B).

## 4. Discussion

NPCD is a rare neurovisceral lysosomal storage disorder characterized by a wide spectrum of clinical phenotypes. However, almost all patients ultimately develop progressive and fatal neurological disease, even if the age at onset of neurological symptoms is extremely variable, ranging from early infancy to adulthood. To date, miglustat is the only approved treatment for the neurological manifestations of the disease in adult and pediatric NPCD patients. Although miglustat does not prevent the development of neurological symptoms [44], experience with hundreds of treated patients over the last decade demonstrates its effectiveness in reducing the rate of disease progression and stabilizing the neurological disease [45,46,47]. However, treatment should be started as soon as the first neurological signs become evident [4,46]. This evidence highlights the crucial role of neurological disease-associated biomarkers to monitor disease progression and optimize care in NPCD patients. 

Several studies have reported an association between CSF levels of NfL and neuronal death and axonal degeneration in various neurodegenerative diseases. Although extremely useful, the downside of the CSF biomarkers is the requirement of invasive sampling. Recently, the development of highly sensitive immunometric methods able to reliable detect low levels of proteins in plasma has opened new opportunities in the field of neurodegenerative disease-associated biomarkers. Indeed, the use of plasma NfL and GFAP levels in patients at different landmarks of multiple sclerosis (NeurofilMS) is currently being studied in a clinical trial [48].

In this work we explored the role of plasma NfL as a possible biomarker in NPCD. 

We showed that plasma levels of NfL are significantly increased in patients affected by NPCD compared with age-matched healthy controls. Furthermore, plasma NfL are significantly higher in NPCD patients presenting neurological disease compared with patients free of neurological involvement. Although these results should be confirmed in larger cohorts, the presented data strongly suggest that plasma levels of NfL might be a potential biomarker of neurological disease in NPCD. This hypothesis is supported by the observation that all NPCD patients, who were either asymptomatic or presented visceral involvement without neurological disease, showed levels of plasma NfL comparable with those found in age-matched controls. Conversely, levels of 3β,5α,6β-triol, a widely used biomarker of NPCD, were increased (above the cut off value) in most of them (75%). Furthermore, no correlation between plasma NfL and 3β,5α,6β-triol was found. These data confirmed a poor correlation between oxysterols levels and neurological disease in NPCD.

It is interesting to look at the results obtained in the two patients affected by the EISL phenotype. These patients presented the highest values of 3β,5α,6β-triol, reflecting the extremely severe visceral disease that led to death within the first year of life. It has been extensively documented that apart from patients affected by this clinical phenotype and exceptional adult cases, all NPCD patients ultimately develop progressive and fatal neurological disease. Therefore, it is possible to hypothesize that even though neurological symptoms are subtle in patients affected by the EISL phenotype, they died from liver failure or respiratory insufficiency before neurological involvement become evident and if they had survived, they probably would have developed severe neurological disease [49]. Indeed, the two patients affected by the EISL phenotype were the only pediatric non-neurological patients displaying levels of plasma NfL that overlapped with those found in neurological patients. However, it is important to acknowledge that EISL patients were 1 and 2 months old, respectively, at the time of NfL assessment, while the pediatric healthy controls were older than 1 year. Therefore, a different behavior due to age could not be ruled out in these patients and further studies should be done to clarify the role of NfL in this particular clinical phenotype.

It should be noted that the highest levels of plasma NfL (581.4 pg/mL) were found in the most severely affected neurological patient in the studied cohort (NP4; Table S1) who presented the EI phenotype and died at 3 years of age, while the lowest value (7.59 pg/mL) was observed in an adult patient diagnosed at 36 years due to mild splenomegaly (NP25; Table S1). Furthermore, two patients developed neurological disease during follow-up and their NfL levels increased 2- and 4-fold, respectively. Although the number of patients is too low to draw definitive conclusion, these data further support the possible association between NfL levels and neurological disease. It would be interesting to follow the development of plasma NfL levels during the further natural course of the NPCD in single patients over months or even years.

It is important to point out the low specificity of plasma NfL levels, which make them unsuitable for NPCD diagnosis, in contrast to their possible high sensitivity and valuable use for assessing and monitoring the neurological course once the diagnosis is definitively established by means of biochemical or molecular methods.

In two patients, samples before and after miglustat treatment were assessed. Both neurological disease and NfL remained stable in one of them while in the other a slightly decrease in NfL levels and an amelioration of symptoms were observed. However, the possible utility of this marker in monitoring the response to therapy remains to be addressed.

## 5. Conclusions

In conclusion, this study shows that plasma levels of NfL are increased in NPCD patients and correlate with the presence of neurological disease, suggesting a possible role of this molecule as a neurological disease biomarker in NPCD. Based on these promising results, studies aimed at evaluating the levels of NfL during long-term follow-up and in response to treatment in larger cohorts should be undertaken.

## Figures and Tables

**Table 1:** Characteristics of the studied cohort.

	Pediatric NPCD Patients (*n* = 12)	Adult NPCD Patients (*n* = 14)
Gender		
Male	6	5
Female	6	9
Clinical phenotype		
EISL	2 *	0
EI	2 (1+)	0
LI	4	0
J	2	4
A	0	6
NC	2	4
Number of analyzed samples		
	15	24
Age at sampling (years)		
Mean	4.8	29.4
Range	0.08–13.08	21.58–53.08
Presence of Neurological involvement at sampling		
	6	14

EISL: early infantile severe lethal; EI: early infantile; LI: late infantile; J: juvenile; A: adult; NC: nonclassified because of lack of neurological involvement at last follow-up [41] Two J patients were twins and 2 adults were siblings, as were 2 J, 1 A, and 2 NC patients. * deceased due to liver failure and respiratory insufficiency, respectively. + deceased at 3 years of age due to neurological disease.

**Table 2:** Plasma levels of NfL (pg/mL).

	Pediatric		Adults	
	Healthy Controls (*n* = 39)	NPCD (*n* = 15)	Healthy Controls (*n* = 36)	NPCD (*n* = 24)
Median	7.94	28.97 ****	11.2	25.6 ****
IQ range	6.38–9.75	15.83–70.21	8.265–15.26	15.9–34.75
Range	4.56–21.2	8.82–581.4	4.01–33.97	7.59–62.5

**** *p* < 0.0001.

**Table 3:** Plasma levels of NfL (pg/mL).

	Pediatric				Adults			
	Healthy Controls (*n* = 39)	NPCD (*n* = 12)	NPCD Neuro (*n* = 4)	NPCD Non Neuro (*n* = 7)	Healthy Controls (*n* = 36)	NPCD (*n* = 14)	NPCD Neuro (*n* = 9)	NPCD Non Neuro (*n* = 5)
Median	7.94	33.07 ****	65.75	19.26 *	11.20	27.02 ***	33.10	16.07 *
IQ range	6.38–9.75	15.78–67.98	47.35–453.60	14.12–38.24	8.20–15.47	14.91–36.18	27.02–39.63	9.51–19.43
Range	4.56–21.20	8.82–581.40	42.70–581.40	8.82–73.50	4.01–33.97	7.59–62.50	11.12–62.50	7.59–21.15

**** *p* < 0.0001; *** *p* < 0.001 NPCD patients vs. healthy age matched controls. * *p* < 0.05 NPCD neurological vs. non-neurological patients.

## Data Availability

The data presented in this study are available on request from the corresponding author. The data are not publicly available due to privacy issues.

## Supplementary Materials

The following are available online at https://www.mdpi.com/article/10.3390/jcm10204796/s1, Table S1: Plasma NfL in individual NPCD patients.
