# Supplementary material for: Plasma Neurofilament Light (NfL) in Patients Affected by Niemann–Pick Type C Disease (NPCD)
Source: J Clin Med. 2021 Oct 19;10(20):4796. doi: 10.3390/jcm10204796 (PMC8537496; doi:10.3390/jcm10204796)
Supplement: Supplementary file 1 [file jcm-10-04796-s001.zip › jcm-1418312-proofed-supplementary.pdf]

Supplementary Table S1: Plasma NfL in individual NPCD patients

Patient code	Gene	Diagnosis	Clinical phenotype	Age at NfL assay (Y)	Neurological sings at NfL assessment	NfL (ng/ml)
NP1	NPC2	Genotyping: pathogenetic variant in homozygosity Filipin: classic phenotype	ESL +	0.08	None	38.24
NP2	NPC1	Genotyping: pathogenetic variant in homozygosity Filipin: ND	ESL +	0.17	None	73.50
NP3	NPC1	Genotyping: 2 pathogenetic variants Filipin: classic phenotype	Late infantile	0.25	None	19.26
NP4	NPC1	Genotyping: pathogenetic variant in homozygosity Filipin: ND	Early infantile +	3.0	Developmental delay, severe hypotonia	581.43
NP5	NPC1	Genotyping: pathogenetic variant in homozygosity Filipin: ND	Early infantile	1.42	Developmental delay	70.21
NP6	NPC1	Genotyping: 2 pathogenetic variants Filipin: classic phenotype	Late infantile	0.42	None	27.90
				5.3	Balance problems, clumsiness	123.2
NP7	NPC1	Genotyping: 2 pathogenetic variants Filipin: ND	Late infantile	3.5	Developmental delay	61.29
NP8	NPC1	Genotyping: 1 pathogenetic variant; 1 VUS Filipin: classic phenotype	Late infantile	12.0	Ophthalmoplegia, dysarthria, dysphagia, intellectual disability, inability to walk	42.70
NP9	NPC1	Genotyping: 1 pathogenetic variant, 1VUS Filipin: variant phenotype	Juvenile	8.5	None	14.12
				9.5	None	15.83
				11.25	Bilateral hands tremor; mirror movements, dystonia	28.97
NP9sib	NPC1	Genotyping: 1 pathogenetic variant, 1VUS Filipin: variant phenotype	Juvenile	13.08	None	15.61

NP11	NPC2	Genotyping: 2 pathogenetic variants Filipin: ND	NC	2.5	None	8.82
NP12	NPC3	Genotyping: 2 pathogenetic variants Filipin: ND	NC	0.92	None	16.30
NP13*	NPC1	Genotyping: pathogenetic variant in homozygosity Filipin: classic phenotype	Juvenile	21.58	Vertical gaze supranuclear ophthalmoplegia	40.44
NP13sib*	NPC1	Genotyping: pathogenetic variant in homozygosity Filipin: ND	Juvenile	21.58	Dysphagia, dysarthria, ataxic gait, vertical gaze supranuclear ophthalmoplegia	29.44
NP15	NPC1	Genotyping: 2 pathogenetic variants Filipin: classic phenotype	Juvenile	21.83	Epilepsy, ataxia, dysarthria dysphagia, vertical supranuclear gaze palsy, cognitive decline	11.11
				22.75		10.50
NP16	NPC1	Genotyping: pathogenetic variant in homozygosity Filipin: classic phenotype	Juvenile	20.75	Gait problems, dysarthria, dysphagia	33.1
NP9sib	NPC1	Genotyping: 1 pathogenetic variant, 1VUS Filipin: variant phenotype	Adult	19.33	None	21.15
				24.83	Mild tremor, memory problems	21.19
NP18	NPC1	Genotyping: 2 pathogenetic variants Filipin: classic phenotype	Adult	20.83	Mild coordination deficit	38.81
				23.42	None	26.6
				24.75	None	27.4
NP18sib	NPC1	Genotyping: 2 pathogenetic variants Filipin: classic phenotype	Adult	26.25	Dystonia, dysphagia, dysarthria, vertical supranuclear gaze palsy	35.3
				27.5		36.6
				28.42		41.1
				31.17		27.4
NP20	NPC1	Genotyping: 1 pathogenetic variant; 1 VUS Filipin: classic phenotype	Adult	37.92	Dysphagia, dysarthria, coordination problems, vertical supranuclear gaze palsy	30.8
NP21	NPC1	Genotyping: 2 pathogenetic variants Filipin: classic phenotype	Adult	37.0	Balance problems, tremors, mild dysphagia	62.5

NP22	NPC1	Genotyping: pathogenetic variant in homozygosity Filipin: ND	Adult	42.92	Cataplexy, dysphagia, dysarthria, ataxia, cognitive decline, myoclonus, vertical supranuclear gaze palsy	24.6
NP9sib	NPC1	Genotyping: 1 pathogenetic variant, 1VUS Filipin: variant phenotype	NC	26.08	None	11.4
				27.17	None	21.92
NP9sib	NPC1	Genotyping: 1 pathogenetic variant, 1VUS Filipin: variant phenotype	NC	29.08	None	16.1
				36.25	None	14.89
NP25	NPC1	Genotyping: 1 pathogenetic variant, 1VUS Filipin: variant phenotype	NC	39.5	None	7.59
				41.17	None	9.94
NP26	NPC2	Genotyping: pathogenetic variant in homozygosity Filipin: ND	NC	53.83	None	17.71

EISL: early infantile severe lethal; EI: early infantile; LI: late infantile; J: juvenile; A: adult; NC: non-classified because of lack of neurological involvement at last follow up [1].

NPCD diagnosis was established by NPC1 and NPC2 genotyping. Biochemical confirmation by filipin staining was done in all patients presenting at least one allele carrying a VUS and whenever available in cases with pathogenetic mutation is both alleles. Patients with at least 1 VUS and presenting a variant biochemical phenotype presented elevated levels of oxysterols and/or or N-palmitoyl-O-phosphocholineserine (PPCS**)**

Siblings within a family were identified with the same NP number followed by sib

*twins; + deceased
